# Optimising Percutaneous Coronary Interventions: The Impact of Stent Type and Diameter on Long-Term Clinical Outcomes in Large Coronary Arteries

**DOI:** 10.3390/medicina60040600

**Published:** 2024-04-04

**Authors:** Gökhan Gök, Murat Akçay, Ufuk Yıldırım, Metin Çoksevim, Korhan Soylu, Mahmut Şahin

**Affiliations:** 1 Clinic of Cardiology, Terme State Hospital, 55139 Samsun, Turkey; drgokhangok@gmail.com; 2 Department of Cardiology, Ondokuz Mayıs University Faculty of Medicine, 55200 Samsun, Turkey; drmuratakcay@hotmail.com (M.A.); ufukyildirim2715@yahoo.com.tr (U.Y.); korhansoylu@yahoo.com (K.S.); drmahmutsahin@gmail.com (M.Ş.)

**Keywords:** coronary artery disease, percutaneous coronary intervention, long-term outcomes

## Abstract

*Background and Objectives:* Our study aimed to reveal the effect of using 4 mm bare-metal stents (BMS), 4 mm drug-eluting stents (DES), or 3 mm DES with 4 mm diameter balloon post-dilation strategies on long-term clinical outcomes and endpoints for large-diameter coronary artery percutaneous coronary intervention (PCI). *Materials and Methods:* In our study, patients who had undergone PCI were retrospectively screened between January 2014 and July 2020. The study included 350 patients and was divided into three groups; Group I (*n* = 134) included patients with direct 4.0 mm BMS implantation, Group II (*n* = 109) included patients with direct 4.0 DES implantation, and Group III (*n* = 107) included patients with 4mm NC post-dilatation after 3 mm DES implantation. Primary endpoints were determined as target lesion revascularisation, cardiac mortality, and myocardial infarction associated with the target vessel. Our secondary endpoint was all-cause mortality. *Results:* No differences were observed between the groups in terms of the baseline variables. Stent length was the highest in Group II and the shortest in Group III. There were no significant differences between the groups regarding major adverse cardiovascular events (MACE). *Conclusions:* Our study suggests that in percutaneous coronary interventions for non-complex lesions, there is no significant difference in MACE outcomes when directly implanting a 4 mm diameter DES, a 4 mm diameter BMS, or a 3 mm diameter DES, followed by post-dilation with an appropriately sized NC balloon when the target vessel diameter is in the range of 4 to 4.4 mm.

## 1. Introduction

In the realm of percutaneous coronary interventions, the evolution from bare-metal stents (BMS) to drug-eluting stents (DES) marked a significant milestone by substantially reducing in-stent restenosis rates. This advantage is particularly pronounced in treating lesions less than 3 mm in diameter or exceeding 15 mm in length, establishing DES as the preferred option in such cases [1,2,3]. Consequently, contemporary guidelines, including those from the National Institute for Health and Clinical Excellence (NICE), advocate for DES usage in coronary arteries smaller than 3 mm or for lesions longer than 15 mm [1,2,3,4]. However, for larger coronary arteries—with diameters exceeding 3 mm—the comparative efficacy of DES over BMS remains ambiguously defined in current literature. Despite recent guidelines universally recommending DES for all lesion types, debates persist regarding their superiority in larger vessels [1,2,3,4,5,6,7,8,9].

Our study introduces a novel perspective by focusing on a cohort rarely emphasised in existing research: patients with large coronary arteries undergoing stent implantation. Specifically, we retrospectively analysed stenting procedures conducted in our clinic over the past six years, focusing on patients who received a 4 mm diameter BMS, a 4 mm diameter DES, or a 3 mm diameter DES followed by a 4 mm non-compliant balloon post-dilation. This unique approach allows us to shed light on the long-term clinical outcomes of stent type and diameter selection in large coronary arteries, an area that remains underexplored in current guidelines and literature. By doing so, we aim to bridge a critical gap in our understanding of optimal stent choice and deployment strategy, thereby informing future guidelines and clinical practice in the treatment of coronary artery disease.

## 2. Materials and Methods

### 2.1. Study Population

**Figure 1:**
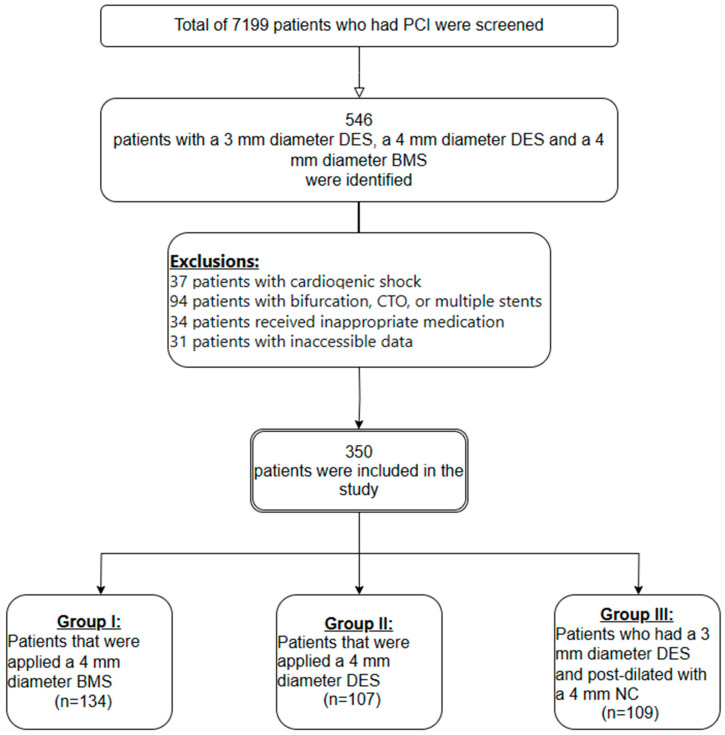
Patient selection and grouping flow chart.

In our study, 7199 patients in total who underwent PCI between January 2014 and July 2020 at Ondokuz Mayis University, Faculty of Medicine, Department of Catheterization Laboratory were screened. A total of 546 patients who had a 4 mm diameter BMS, a 4 mm diameter DES, or a 3 mm diameter DES with a 4 mm NC post-dilatation were selected among these patients. Among these patients, the patients in which the stent was expanded to a diameter of 4.0–4.4 mm were included in the study. A total of 196 patients were excluded from the study since 37 patients had cardiogenic shock, 94 patients had bifurcation, CTO, LMCA, and multiple stent interventions, 34 patients had not received the planned antiplatelet treatment, 7 patients had less than six months follow-up, and the information of 31 patients could not be obtained. The study covered a total of 343 patients with the following characteristics: patients who had a 4.0 mm diameter BMS (*n* = 134, Group I), patients who had a 4 mm diameter DES (*n* = 104, Group II), and patients who had a 3 mm diameter DES and who underwent a 4 mm diameter NC balloon post-dilation (*n* = 105, Group III) (Figure 1). The post-dilatation strategy using a 3 mm DES and a 4 mm non-compliant balloon applied to vessels with a diameter above 3 mm was not reimbursable according to the health practice regulation performed in our country. In patients for whom a 4.0 mm DES was preferred, the cost of stent was covered by the patient. The 4 mm diameter BMS used in our study were Ephesus-2 stents produced by the Alvimedica company. Twenty-two of the 4 mm diameter DES (20%) were Evorolimus-eluting stents produced by the Boston company, and eighty-five (80%) were Evorolimus-eluting stents produced by the Abbott company. Twenty-three of the 3 mm diameter DES (21%) were Evorolimus-eluting stents produced by the Boston company, and eighty-six (79%) were Evorolimus-eluting stents produced by Abbott company.

The following exclusion criteria were determined: bifurcation interventions, CTR interventions, multiple stent interventions, cases with stent expansion above 4.4 mm with high pressure during post-dilation, patients who underwent post-dilation with a balloon of 4.5 mm or more, inappropriate pre-medications, not receiving anti-platelet therapy within the recommended time for any reason, the presence of cardiogenic shock, and the use of 3.5 mm diameter stents.

Information related to the characteristics of the patients and the procedure was obtained from hospital records via the e-pulse system or by contacting patients over the phone. The mortality information for the patients was accessed using the death notification system of the Ministry of Health and by contacting the relatives of the patients over the phone. The study was approved by the ethics committee of the Faculty of Medicine, Ondokuz Mayis University (No: 2020/359), and adhered to the Declaration of Helsinki (2013 version).

Basal demographic characteristics, age, gender, diabetes mellitus as a risk factor, hypertension, cigarette use, family history, previous myocardial infarction, coronary artery bypass graft, and history of previous percutaneous coronary intervention were obtained by scanning the data records. Also, laboratory values were retrospectively scanned, and creatinine, haemoglobin, LDL cholesterol values, and echocardiographic left ventricular ejection fraction (LVEF) values were obtained. Before or after the procedure, these data were recorded as the values observed during patients’ hospital stay.

### 2.2. Angiographic Analysis

The locations and characteristics of the lesions (the vessel where the lesion was located, whether there was CTR, whether there was bifurcation, whether multiple stenting was performed) were examined by accessing the angiographic records of the patients. Before or during the procedure, all the patients’ pre-medications were recorded. The diameter, length, and type of the stent applied to the patients were selected according to the operator’s decision. As an arterial approach to the patients enrolled in the study, angiography was performed with femoral or radial routes. Although pre-dilatation and post-dilatation were optional, post-dilatation was performed in all patients in Group III.

### 2.3. Definitions

Target-vessel revascularisation (TVR) was defined as a requirement of PCI or CABG, again along the entire vessel in the proximal and distal part of the target lesion.

MI associated with the target vessel was defined as the acute coronary syndrome associated with the proximal or distal part of the target lesion.

Target lesion revascularisation (TLR) was defined as the requirement of PCI or CABG due to restenosis or thrombosis covering the 5 mm proximal part or 5 mm distal part of the target lesion.

Stent thrombosis was defined as the formation of clots in the stent after stent insertion. It was classified as acute (first 24 hours after insertion of the stent into the vessel), early (first month), late (first year) or very late (after one year). Definite stent thrombosis is an angiographically or pathologically verified thrombosis, and in our study, definite stent thrombosis was examined [1,2,3,4,5,6,7,8,9,10].

Major haemorrhage was defined as bleeding that required three units or more of blood transfusions in 24 h [8,9,10].

### 2.4. Statistical Analysis

Statistical analyses were carried out using IBM SPSS V21. (SPSS Inc., Chicago, IL, USA) The normality distributions of quantitative data were performed using the Kolmogorov–Smirnov test. In order to compare the data that meets the normal distribution, the ANOVA test was used, and in order to compare the data that did not suggest a normal distribution, the Kruskal–Wallis test was used. Pearson’s chi-square test was applied to compare qualitative data. Survival analyses were performed using the Kaplan–Meier test. The data were presented as *n* (%), mean ± standard deviation and median (minimum–maximum). The statistical significance value was accepted as *p* < 0.05.

## 3. Results

When the basal demographic characteristics of the patients were examined, there was no distinction among the groups in terms of age, gender, diabetes mellitus as a risk factor, hypertension, cigarette use, family history, prior myocardial infarction, coronary artery bypass graft, and prior percutaneous coronary intervention (Table 1). When the diagnoses of the patients included in the study were considered during the application period, STEMI (*n* = 85) was the most common in the patients after NSTEMI (*n* = 214), but there was no statistically important difference among the groups. When retrospective laboratory values were examined, there was no statistical difference between the groups in terms of LDL-cholesterol, creatinine, and haemoglobin; the mean LDL value of all patients was 109 ± 41.2 mg/dL. When the retrospective echocardiographic data were examined, the comparison of the groups suggested that the mean EF value in Group I was 50% (25–70), in Group II was 53% (23–66), and in Group III was 54% (30–72), and a statistically important difference was detected between Group I and Group II, and Group I and Group III (*p* < 0.001). The proportion of patients with an EF of >40% was found to be 85% in Group I, 86.8% in Group II, and 94.5% in Group III, and there was a trend towards statistical significance between the groups. Particularly, the proportion of patients with >40% EF in Group III was numerically higher than in Groups I and II (*p* = 0.058) (Table 1).

RCA (*n* = 183, 52.3%), then LAD (*n* = 83, 23.7%), and CX (*n* = 50, 14.3%) were the most common locations in the patients who were covered in the study. There was no statistical distinction among the groups in terms of other lesions (Table 2). When we compared the groups, ostial lesions were most commonly observed in Group II (15.9%), then in Group I (12.7%), and finally in Group III (8.3%), and there was no statistically significant difference between the groups (*p* = 0.229) (Table 2). When we compared the groups according to the post-PCI TIMI flows, TIMI 3 flows were obtained at a rate of 88.8% in Group I, 93.5% in Group II, and 89.9% in Group III, and there was no statistically important distinction among groups (Table 2). Again, when the groups were compared in terms of the duration of DAPT used in patients, no significant difference was determined. When the use of OAC was studied, it was found that 3.4% of patients used OAC, and no distinction among the groups was determined (Table 2).

When the mean stent lengths used for PCI were studied, these were 18 (6–48) mm in Group I, 20 (8–38) mm in Group II, and 23 (15–48) mm in Group III (*p* < 0.001). The stent length in Group I was significantly lower than in both Group II and Group III patients (*p* < 0.001). In the study, 4 mm diameter stents were used in Group I and Group II, 3 mm diameter stents were used in Group III and post-dilatation was performed with a 4 mm diameter NC balloon in patients who underwent a post-dilatation. When we examined the final stent diameters among the groups, Group I and Group II stents reached a mean final diameter of 4.2 mm, while Group III stents reached a mean final diameter of 4.1 mm (Table 3).

When the cardiac mortality rates and times of cardiac mortality were compared, no statistically important differences were determined among the groups. The median follow-up period of all patients was 36 (6–75) months. When the follow-up periods were examined, Group I patients were followed up for median of 48 (6–75) months and there was a statistically important distinction between Group I and Group II, and Group I and Group III (*p* < 0.045). When the all-cause mortality rates and the all-cause mortality times were compared during this follow-up period, no statistically important distinction was determined among the groups (Table 4).

Of the 343 patients included in the study, target-vessel–related MI was detected in 12 (3.4%) patients and target-lesion revascularisation (TLR) was detected in 7 (2%) patients during the follow-up period; no statistically important distinction was determined among the groups. Stent thrombosis was observed in only one patient in Group I and there was no statistically important distinction among the groups (*p* = 0.646). Again, of 343 patients, 14 (4%) patients had MI associated with other coronary vessels during follow-up, and there was no significant statistical difference between the groups in terms of MI and major haemorrhage rates associated with other coronary vessels. When the complication times were examined, no statistically important distinction was determined among the groups in terms of the time of MI associated with the target vessel, the time of target-lesion revascularisation, and the time of myocardial infarction associated with other coronary vessels (Table 4).

## 4. Discussion

In our study, we explored the clinical outcomes of DES versus BMS in large coronary arteries, as well as the effects of stent oversizing with a non-compliant balloon post-dilation. Our findings reveal no significant difference in thrombosis, TLR, MI, long-term cardiac mortality, or all-cause mortality between the stent types in these scenarios. Notably, DES with a diameter of 3 mm, capable of expanding to 4.4 mm through post-dilation, were associated with favourable cardiac endpoints, akin to 4 mm diameter stents, when optimally post-dilated.

While the importance of DES is no longer discussed in the guidelines, the aim of our retrospective study was to point out the unclear predominance of DES in large vessels over BMS, and the differences in the literature. The significant predominance of DES over BMS has been demonstrated in many randomised trials and meta-analyses in the case of stent restenosis [10,11,12,13]. The absence of significant differences in clinical outcomes for the use of DES and BMS in large vessels, the inclusion of cost-efficiency analyses, and the evaluation of it in terms of accessibility suggest that discussions will intensify in the future. In Basket’s study, the use of DES in small vessels (<3 mm) and long lesions (>15 mm) was found to be cost-effective [2,6]. In a study by Steinberg et al. [14] involving 466 patients, they compared the administration of BMS with DES to ≥3.5 mm coronary artery stenoses. No difference was determined between the use of BMS and DES at the end of one year in terms of target-lesion revascularization.

In BMS patients, in-stent restenosis (ISR) occurs after a median of 90 days after STEMI and a median of 125 days after non-STEMI [15,16]. In a study that evaluated 39 patients with DES-associated ISR, the mean ISR duration was detected to be approximately 12 months [15,16,17]. Due to the anti-proliferative effects of drugs, ISR may occur approximately nine months later in DES patients than BMS. In previous studies, the length of the stent, the diameter of the stent, diabetes mellitus, renal failure, and the interval between two stents have been determined as risk factors for ISR. Furthermore, a one-millimetre rise in stent length was related to a 2% higher risk of ISR, and a one-millimetre rise in stent diameter was related to a 76% lower risk of ISR. This shows that even in patients requiring exclusively big coronary stents, there is a noteworthy relationship among both stent diameter and stent length with ISR. In large-diameter vessels, comparative studies based on the benefits of BMS or DES selection, based on stent thrombosis, cost-effectiveness, and ease of access to DES have been carried out [18,19,20,21,22].

When the two-year follow-up results of the study by Hyun-Tae Kim et al. [7] performed with 304 patients, which compared DES and BMS in patients with 4 mm diameter stent insertion were investigated, no important distinction was observed among the groups in terms of cardiac mortality, MI, stent thrombosis, and TVR^1^. In the study carried out by Ming-JerHsieh et al. [9] with 1096 patients, patients with lesions in vessels 3 mm and above to which PCI was administered were divided into four different groups in terms of their vessel diameters, such as 3–3.25 mm, 3.26–3.5 mm, 3.51–3.75 mm, and 3.76–4.5 mm; in each group, they were further divided into those who had been administered DES or BMS, and were included in the study [1,7,19,20,21,22,23]. When the first three groups were considered, no important distinction was determined in terms of cardiac mortality, MI, and stent thrombosis rates, and only TVR was found to be predominant to BMS. In group four, that is, in patients with vessel diameters of 3.76–4.5 mm, DES lost its advantage against BMS at all of these endpoints. As a result, this study suggests that DES lose their predominance over BMS after 3.76 mm. The results of our study support these studies and show that DES with a diameter of 3 mm can be used in coronary arteries with a diameter of 4–4.4 mm by properly post-dilating them. The differences between Ming-JerHsieh et al.’s study compared to our study are the inclusion of CTR, bifurcation lesions, and multiple stent interventions [1,9,20,21,22,23]. In the study by Hyun-Tae Kim et al., CTR and multiple stent interventions were excluded, and bifurcation lesions were included in the study [1,7,9]. In our study, we excluded bifurcation procedures, multiple stent interventions, and patients with CTR lesions in order to minimise the need for advanced operator experience, differences in technical procedures that were performed, and bias between stents.

EF level, which is one of the main characteristics of our study, was found to be importantly lower in Group I than in other groups. Similarly, the follow-up period was importantly longer in Group I than in the other groups. Despite being insignificant proportionally, the excess of cardiac events in Group I might be related to these two parameters. However, although the mean stent length in patients in Group I was significantly shorter than the other groups, we postulate that the follow-up cardiac events in Group I was due to the reducing effect of drug release on the presence of cardiac events.

Recent guidelines recommend DES to all patients, regardless of the diameter of the vessel. When the results of our study are examined, a well–post-dilated 3 mm diameter DES or a 4 mm diameter BMS show similar results to a 4 mm diameter DES, with the condition that the final stent diameter is 4.0–4.4 mm. However, it is necessary to know the stents in the laboratory well and to choose the recommended diameter by knowing the maximum expansion values. DES with a 3 mm diameter in our laboratory had the ability to be expanded up to 4.4 mm, and they were used as needed [1,19,20,21,22,23].

Recent literature presents divergent findings regarding stent selection in the management of large coronary artery lesions. Changal et al. reported that the application of new-generation DES is associated with enhanced outcomes in coronary arteries with ≥3 mm diameter [24]. Conversely, Singhal et al. posited that the utilization of BMS could be deemed suitable for coronary arteries with a diameter of ≥3.5 mm. This further increases the importance of our study [25].

The findings of this retrospective analysis, while shedding light on the comparative outcomes of DES and BMS in large coronary arteries, necessitate cautious interpretation. Given the study’s retrospective design and the inherent limitations associated with our sample size, the results presented herein cannot be extrapolated to the broader patient population with absolute certainty. It underscores the imperative need for individualised patient care, where decisions regarding stent selection are tailored to the unique clinical profiles of each patient, taking into account not only the anatomical and physiological considerations but also the potential influence of racial and regional factors on disease manifestation and treatment efficacy. Therefore, the generalisation of our findings should be approached with prudence, reinforcing the call for further prospective studies to validate these observations and guide clinical practice on a more individualised basis.

### Study Limitations

Our study is a non-randomised, monocentric, and retrospective study. In our study, EF levels, lengths of stents inserted, and follow-up durations varied significantly between the groups. At the same time, the number of interventions carried out on the primary coronary lesions also differed considerably between the groups. Stent type and size selection were left to the discretion of the operator, and intravascular imaging techniques were not employed. These parameters were major limitations of our study.

## 5. Conclusions

In percutaneous coronary interventions, single-stent interventions are carried out on lesions that do not require bifurcation, CTO, or multiple stent administration; when the vessel is in the range of 4–4.4 mm, no significant difference is determined in terms of TLR, target-vessel–associated MI, cardiac mortality, and all-cause–related mortality in terms of inserting a 4 mm diameter DES, a 4 mm diameter BMS, or a 3 mm diameter DES and optimised post-dilatating with a 4 mm diameter balloon.

## Figures and Tables

**Table 1:** Distribution of baseline demographic parameters between the groups.

		Group I (4 mm BMS) (*n* = 134) Mean ± SD Median (min–max.)	Group II (4 mm DES) (*n* = 105) Mean ± SD Median (min–max.)	Group III (3 mm DES, 4 mm NC) (*n* = 104) Mean ± SD Median (min–max.)	*p* Value
Age (year)		62 ± 10.8	61 ± 10.1	62 ± 9.8	0.653
Gender	Men, *n* (%)	116 (86.6)	88 (83.2)	86 (82.6)	0.648
	Women, *n* (%)	18 (13.4)	17 (16.8)	18 (17.4)	
Diabetes mellitus, *n* (%)		35 (26.1)	33 (30.8)	4 (31.2)	0.617
Hypertension, *n* (%)		69 (51.5)	54 (50.5)	51 (46.8)	0.614
Cigarette, *n* (%)		69 (51.5)	57 (53.3)	52 (47.7)	0.543
Family history, *n* (%)		41 (30.6)	31 (29)	25 (22.9)	0.390
Previous MI, *n* (%)		39 (29.1)	27 (25.2)	34 (31.2)	0.515
Previous PCI, *n* (%)		25 (18.7)	15 (14)	28 (25.7)	0.092
Previous CABG operation, *n* (%)		15 (11.2)	12 (11.2)	9 (8.3)	0.703
STEMI		37 (27.61)	22 (20.56)	25 (23.85)	0.444
NSTEMI		82 (61.2)	66 (62.6)	63 (59.6)	0.904
Stable CAD		15 (11.19)	17 (16.82)	16 (16.51)	0.369
LDL (mg/dL)		109 ± 35.5	107 ± 45.6	112 ± 43.7	0.645
Creatinine (mg/dL)		1.0 ± 0.3	1.1 ± 1.2	1.1 ± 1.1	0.519
Haemoglobin (gr/dL)		13.7 ± 1.9	13.7 ± 2.1	13.8 ± 1.7	0.866
EF (%)		50 (25–70)	53 (23–66)	54 (30–72)	<0.001
EF (%)	<%40, *n* (%)	20 (15)	13 (13.1)	6 (5.5)	0.058
	≥%40, *n* (%)	114 (85)	92 (86.9)	98 (94.5)	

Quantitative variables were specified as mean ± SD. Categorical variables were shown as number and percentage values. MI: myocardial infarction; PCI: percutaneous coronary intervention; CABG: coronary artery bypass graft surgery; STEMI: ST-segment elevation myocardial infarction; NSTEMI: Non–ST-segment elevation myocardial infarction; CAD: coronary artery disease; LDL: low-density lipoprotein; EF: ejection fraction.

**Table 2:** Coronary lesion characteristics, TIMI flow grade, and medications between the groups.

		Group I (4 mm BMS) (*n* = 134) Mean ± SD Median (min–max.)	Group II (4 mm DES) (*n* = 105) Mean ± SD Median (min–max.)	Group III (3 mm DES, 4 mm NC) (*n* = 104) Mean ± SD Median (min–max.)	*p* Value
LAD, *n* (%)		23 (17.2)	27 (25.2)	33 (30.3)	0.052
CX, *n* (%)		21 (15.7)	15 (14)	14 (12.8)	0.818
RCA, *n* (%)		79 (59)	49 (45.8)	55 (50.5)	0.114
Saphenous graft, *n* (%)		6 (4.5)	3 (2.8)	3 (2.7)	0.697
Osteal lesion	Yes	17 (12.7)	17 (15.9)	9 (8.3)	0.229
	No	117 (87.3)	90 (84.1)	100 (91.7)	
TIMI 1, *n* (%)		5 (3.7)	2 (1.9)	3 (2.7)	0.687
TIMI 2, *n* (%)		10 (7.5)	5 (4.7)	8 (7.3)	0.786
TIMI 3, *n* (%)		119 (88.8)	100 (93.5)	98 (89.9)	0.702
Dual antiplatelet duration (months)		12 (1–24)	12 (3–36)	12 (1–24)	0.501
Oral anticoagulant using *n,* (%)	Yes	5 (3.7%)	1 (0.9%)	6 (5.5%)	0.175
	No	129 (96.3%)	106 (99.1%)	103 (94.5%)	

Quantitative variables were specified as mean ± SD. Categorical variables were shown as number and percentage values. TIMI: thrombolysis in myocardial infarction; LAD: left anterior descending, CX: circumflex; RCA: right coronary artery.

**Table 3:** Coronary stent, non-compliant balloon size, and type characteristics in the groups.

	Group I (*n* = 134) Median (min–max)	Group II (*n* = 105) Median (min–max)	Group III (*n* = 104) Median (min–max)	*p* Value
Stent length (mm)	18 (6–48)	20 (8–38)	23 (15–48)	**<0.001 ^a,b^**
Stent diameter (mm)	4 (4–4)	4 (4–4)	3 (3–3)	**<0.001 ^b,c^**
NC balloon diameter (mm)	4 (4–4)	4 (4–4)	4 (4–4)	1.000
Final stent diameter (mm)	4.2 (4–4.4)	4.2 (4–4.4)	4.1 (4–4.4)	0.987

^a^: Between Group I and Group II, *p* < 0.001; ^b^: Between Group I and Group III, *p* < 0.001; ^c^: Between Group II and Group III, *p* < 0.001.

**Table 4:** Comparison of groups in terms of primary and secondary outcomes, follow-up times, and mortality rates.

	Group I (4 mm BMS) (*n* = 134) Mean ± SD Median (min–max.) *	Group II (4 mm DES) (*n* = 105) Mean ± SD Median (min–max.) *	Group III (3 mm DES, 4 mm NC) (*n* = 104) Mean ± SD Median (min–max.) *	*p* Value
Target-vessel–associated MI, *n* (%)	6 (4.5)	4 (3.7)	2 (1.8)	0.519
Target-lesion revascularization, *n* (%)	4 (3)	2 (1.9)	1 (0.9)	0.516
Stent thrombosis, *n* (%)	1 (25)	0 (0)	0 (0)	0.646
MI associated with other vessels, *n* (%)	4 (3)	4 (3.7)	6 (5.5)	0.600
Major bleeding, *n* (%)	2 (1.5)	1 (0.9)	0 (0)	0.452
Time to target-vessel–associated MI (months)	22 (1–36)	13 (1–24)	21 (6–36)	0.609
Time to target-lesion revascularization (months)	30 (1–40)	13 (1–24)	6 (6–6)	0.558
Time to MI associated with other vessels (months)	32 (6–48)	12 (12–24)	16 (5–36)	0.385
Cardiac death, *n* (%)	5 (3.7)	2 (1.9)	3 (2.7)	0.687
All-cause death, *n* (%)	10 (7.5)	6 (5.6)	6 (5.5)	0.473
Follow-up period (months)	48 (6–75)	38 (7–54)	33 (6–60)	<0.045 *
Time to cardiac death (months)	16 (6–36)	11 (4–18)	15 (4–24)	0.695
Time to death from all causes (months)	18 (6–46)	18 (3–24)	20 (4–59)	0.621

* Group I and Group II and Group I Group III. MI: myocardial infarction.
